# Imaging of benign salivary gland tumors under the fifth WHO classification, with a focus on rare histologic types and newly listed entities—Part II

**DOI:** 10.1007/s11604-026-02055-w

**Published:** 2026-07-22

**Authors:** Tohru Takeshita, Taro Shimono, Hirotaka Takita, Daiju Ueda, Kayo Ueda, Yukio Miki, Akira Yamamoto

**Affiliations:** 1 Department of Radiology, Osaka Habikino Medical Center, 3-7-1, Habikino, Habikino, Osaka City, 583-8588 Japan; 2 Department of Diagnostic and Interventional Radiology, Graduate School of Medicine, Osaka Metropolitan University, 1-4-3, Asahi-Machi, Abeno-ku, Osaka, 545-8585 Japan; 3 Department of Artificial Intelligence, Graduate School of Medicine, Osaka Metropolitan University, Osaka, Japan; 4 Department of Pathology, Osaka Habikino Medical Center, 3-7-1, Habikino, Habikino, Osaka, 583-8588 Japan

**Keywords:** Salivary gland tumors, WHO classification, Diagnostic imaging, Magnetic resonance imaging, Computed tomography

## Abstract

The World Health Organization (WHO) Classification of Head and Neck Tumors was updated from the fourth edition (published in 2017) to the fifth edition (published in 2024). The current WHO classification lists 15 benign and 21 malignant epithelial salivary gland tumors (SGTs). Among the benign epithelial tumors, four entities are newly listed: intercalated duct adenoma/hyperplasia, striated duct adenoma, sclerosing polycystic adenoma, and keratocystoma. This second article in a two-part review series summarizes the four newly listed benign SGTs, focusing on their classification background, clinicopathologic features, and reported imaging findings. As the published imaging literature remains limited, radiologic-pathologic correlation remains essential for recognizing these rare entities and avoiding overinterpretation of benign lesions. The aims of this review are to clarify the currently recognized imaging features of these rare tumors while emphasizing the need for careful integration of imaging, histopathologic, immunohistochemical, and molecular findings.

## Introduction

Numerous histologic tumor types arise in the salivary glands. Their diversity, rarity, complex terminology, and overlapping microscopic features make the diagnosis of salivary gland tumors (SGTs) challenging for both pathologists and radiologists.

The current World Health Organization (WHO) classification lists 15 benign and 21 malignant epithelial SGTs [1]. Among the benign epithelial tumors, four entities are newly listed: intercalated duct adenoma/hyperplasia (IDA/IDH), striated duct adenoma (SDA), sclerosing polycystic adenoma (SPA), and keratocystoma (KC) [1–4] (Table 1).

**Table 1:** Epithelial salivary gland tumor types in the fifth edition of the World Health Organization Classification of Head and Neck Tumors

Benign epithelial tumors	Malignant epithelial tumors
Pleomorphic adenoma	Mucoepidermoid carcinoma
Basal cell adenoma	Adenoid cystic carcinoma
Warthin tumor	Acinic cell carcinoma
Oncocytoma	Secretory carcinoma
Salivary gland myoepithelioma	Microsecretory adenocarcinoma*
Canalicular adenoma	Polymorphous adenocarcinoma
Cystadenoma of the salivary glands	Hyalinizing clear cell carcinoma
Ductal papillomas	Basal cell adenocarcinoma
Sialadenoma papilliferum	Intraductal carcinoma
Lymphadenoma	Salivary duct carcinoma
Sebaceous adenoma	Myoepithelial carcinoma
Intercalated duct adenoma and hyperplasia*	Epithelial-myoepithelial carcinoma
Striated duct adenoma*	Mucinous adenocarcinoma*
Sclerosing polycystic adenoma*	Sclerosing microcystic adenocarcinoma*
Keratocystoma*	Carcinoma ex pleomorphic adenoma
	Carcinosarcoma of the salivary glands
	Sebaceous adenocarcinoma
	Lymphoepithelial carcinoma
	Squamous cell carcinoma
	Sialoblastoma
	Salivary carcinoma, NOS, and emerging entities

Asterisks (*) indicate entities newly listed in the salivary gland section of the fifth edition of the World Health Organization Classification of Head and Neck Tumors

Abbreviation: NOS, Not otherwise specified

Adapted from the WHO Classification of Tumours Editorial Board [1]

This article is the second part of a two-part series on benign SGTs in the fifth WHO classification. Whereas Part I reviewed rare histologic types, this article focuses on newly listed benign tumors, emphasizing clinical presentation, histopathological features, and imaging findings.

## Genetic alterations in benign SGTs and changes in the WHO classification

Knowledge of the molecular features of SGTs has substantially expanded in recent years [5–20]. Advances in molecular pathology have helped define the disease concepts of the four newly listed benign tumors discussed in this review.

SDA and KC harbor characteristic genetic alterations, and recognition of these alterations strongly supported their classification as distinct entities [19, 20]. In contrast, SPA and IDA/IDH were previously listed under non-neoplastic epithelial lesions in the fourth edition of the WHO classification but were moved to the benign tumor section in the fifth edition [1–4, 21, 22].

**Fig. 1:**
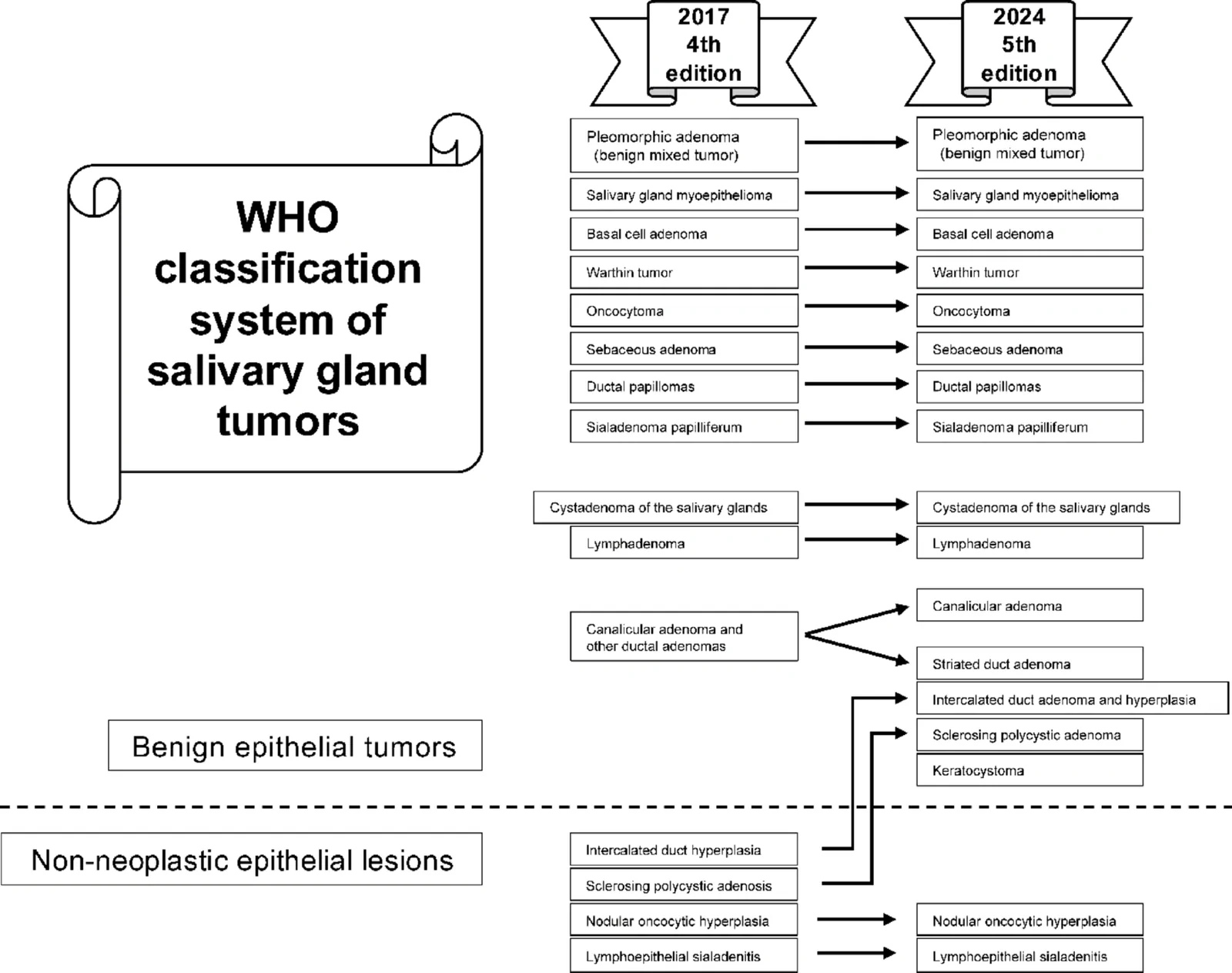
Changes in the WHO classification of salivary gland tumors from the fourth edition to the fifth edition. The diagram illustrates changes in tumor entities from the fourth edition (published in 2017) to the fifth edition (published in 2024). Adapted from the study by Żurek et al. [22]. Abbreviation: WHO, Word Health Organization

Figure 1 summarizes the changes in the WHO classification from the fourth edition to the fifth edition. Table 2 summarizes tumor-specific genetic alterations and corresponding surrogate immunohistochemical markers in benign SGTs.

**Table 2:** Tumor-type-specific genetic alterations in benign salivary gland tumors and corresponding surrogate immunohistochemical markers

Tumor type	Genetic alterations	Surrogate immunohistochemical markers	Note	References
Pleomorphic adenoma	*PLAG1* rearrangement (> 50%)	PLAG1	Primarily expressed in the nuclei of myoepithelial cells	[6]
Pleomorphic adenoma	*HMGA2* rearrangement (10–20%)	HMGA2	Primarily expressed in the nuclei of myoepithelial cells	[7]
Basal cell adenoma, (mostly with tubular and tubulotrabecular growth patterns)	*CTNNB1* gain-of-function mutation (Mostly p.I35T) (50–80%)	β-catenin	Nuclear expression is interpreted as positive Approximately 20% of basal cell nuclei are positive High sensitivity and specificity have been reported	[8–11]
Basal cell adenoma (some cases with membranous growth pattern)	*CYLD* mutation (36%)	–	Associated with Brooke–Spiegler syndrome	[12]
Basal cell adenoma	*FBXW11* missense mutation (p.F517S) (16%)	–	This mutation is mutually exclusive with the *CTNNB1* p.I35T gain-of-function mutation; together, these two mutations account for 94% of BCA cases	[13]
Sialadenoma papilliferum (classic type)	*BRAF* p.V600E mutation (70–100%)	BRAF V600E mutation-specific staining	Both exophytic squamous and endophytic ductal components show positive cytoplasmic staining	[14, 15]
Striated duct adenoma	*IDH2* mutation (Mostly p.R172X) (100%)	IDH1/IDH2 mutation-specific staining	Cytoplasmic granular expression is interpreted as positive	[19]
Keratocystoma	*RUNX2* rearrangement (*IRF2BP2::RUNX2* fusion) (100%)	–	–	[20]

Abbreviations: BCA, basal cell adenoma; BRAF, b-raf proto-oncogene; CTNNB1, catenin beta 1; CYLD, cylindromatosis; FBXW11, F-box and WD repeat domain containing 11; HMGA2, high mobility group AT-hook 2; IDH, intercalated duct hyperplasia; IDH1, isocitrate dehydrogenase 1; IDH2, isocitrate dehydrogenase 2; IRF2BP2, interferon regulatory factor 2 binding protein 2; PLAG1, pleomorphic adenoma gene 1; RUNX2, RUNX family transcription factor 2

Adapted from WHO Classification of Tumours Editorial Board [1]

## Newly listed benign tumors

The clinicopathologic features of the four newly listed benign SGTs are summarized in Table 3, and their key imaging findings are summarized in Table 4.

**Table 3:** Clinicopathologic features of newly listed benign salivary gland tumors

Features	Intercalated duct adenoma/hyperplasia	Striated duct adenoma	Sclerosing polycystic adenoma	Keratocystoma
Age and sex	19–80 years (mean: 53.8 years) M:F ratio 1:1.7	23–81 years (peak in 7th decade of life) No sex predilection	7–84 years (peak in 4th decade of life) M:F ratio 1:1.3 Slight female predominance	1–70 years (median: 34 years) No sex predilection
Location	Parotid gland (Mostly)	Parotid gland (Mostly)	Parotid gland (Mostly)	Parotid gland
Clinical appearance	Incidentally discovered on histopathology (majority)	Parotid mass Submucosal nodule in minor salivary gland location	Parotid mass Submucosal nodule in minor salivary gland location	Parotid mass
Tumor border and Encapsulation	IDA: well-circumscribed, fibrous capsule (+) IDH: ill-defined, fibrous capsule (−)	Well-circumscribed, fibrous capsule (+)	Well-circumscribed, fibrous capsule (−)	Unencapsulated, fibrous capsule (−)
Macroscopic features and size	Usually not visible < 5 mm	Solid 0.5–30 mm (mean: 15 mm)	Solid 10–120 mm (mean: 30 mm)	Multicystic 8–40 mm (median: 25 mm)
Histopathology and growth pattern	Closely packed ductal proliferation set in little stroma	Closely packed ductal proliferation set in little stroma	Solid or solid and cystic Dense sclerotic stroma Ductal formation Proliferation of ductal, myoepithelial, and acinar cells Intraductal epithelial proliferation Inflammatory cell infiltrate	Multicystic Cystic spaces lined by bland-looking keratinizing squamous epithelium
Cytomorphology	Small cuboidal ductal cells (bland) Eosinophilic to amphophilic Scant cytoplasm	Columnar ductal cells (bland) Eosinophilic Cytoplasmic striations	Ductal cells: variable, often apocrine cells Acinar cells containing hyper-eosinophilic granules Myoepithelial cells (intact)	Squamous (bland)
Ductal formation	(+) Prominent, closely packed	(+) Prominent, closely packed	(+) Variable sizes and numbers, haphazardly distributed	(+) Squamous-lined cystic spaces filled with lamellated keratin
Solid tumor nest	(−)	(−)	(−)	(+) Nests of squamous cells
Biphasic ductal and myoepithelial differentiation	(+) Usually difficult to detect on standard HE staining, requiring immunohistochemical staining to highlight myoepithelial cells	(−) If present, only a few basal cells	( +)	(−)
Stroma	Scant	Scant but rich vascular network	Abundant Hyalinized and collagenous Lipomatous (uncommon) Inflammatory cell infiltrate	Present Fibrous Inflammatory cell infiltrate
Characteristic immunohistochemical staining	Specific myoepithelial markers (+) in outer myoepithelial cells S-100 (+) in inner ductal cells	Specific myoepithelial markers (mostly −) S-100 (diffuse +) IDH1/2 mutation-specific immunohistochemical staining ( +)	Specific myoepithelial markers (+) in outer myoepithelial cells	Squamous epithelial markers ( +) Specific myoepithelial marker (−)
Tumor-specific gene alterations	*CTNNB1* mutation *HRAS* mutation (subset)	*IDH2* mutation	PIK3-Akt pathway alterations (usually *PIK3CA* mutations) *PTEN* loss in ductal cells and acinar cells	*RUNX2* rearrangement *IRF2BP2::RUNX2* fusion (majority)

Abbreviations: CTNNB1, catenin beta 1; H&E, hematoxylin and eosin; HRAS, Harvey rat sarcoma viral oncogene homolog; IDA, intercalated duct adenoma; IDH, intercalated duct hyperplasia; IDH1, isocitrate dehydrogenase 1; IDH2, isocitrate dehydrogenase 2; IRF2BP2, interferon regulatory factor 2 binding protein 2; M:F, male-to-female; PIK3-Akt, phosphatidylinositol 3-kinase–Akt; PIK3CA, phosphatidylinositol-4,5-bisphosphate 3-kinase catalytic subunit alpha; PTEN, phosphatase and tensin homolog; RUNX2, RUNX family transcription factor 2. Symbols: ( +), present/positive; (−), absent/negative

**Table 4:** Key imaging findings of newly listed benign salivary gland tumors in the fifth WHO classification

Tumor type	Key imaging findings
Sclerosing polycystic adenoma	Variable imaging appearance Well-circumscribed mass that may appear solid or solid with microcystic foci Low signal intensity on T2-weighted MRI Focal fat attenuation on CT or fat signal intensity on MRI within the mass, uncommon No visible capsule on CT or MRI
Keratocystoma	Multicystic mass Multiple small ring-like enhancing foci within the mass on contrast-enhanced CT Heterogeneous low-to-high signal intensity on T2-weighted MRI No visible capsule on CT or MRI
Striated duct adenoma	Well-circumscribed mass Intense enhancement on contrast-enhanced CT Intratumoral hemorrhage
Intercalated duct adenoma/hyperplasia	Usually not visible on imaging

Abbreviations: CT, computed tomography; MRI, magnetic resonance imaging; WHO, World Health Organization

### SPA

#### Definition

**Fig. 2:**
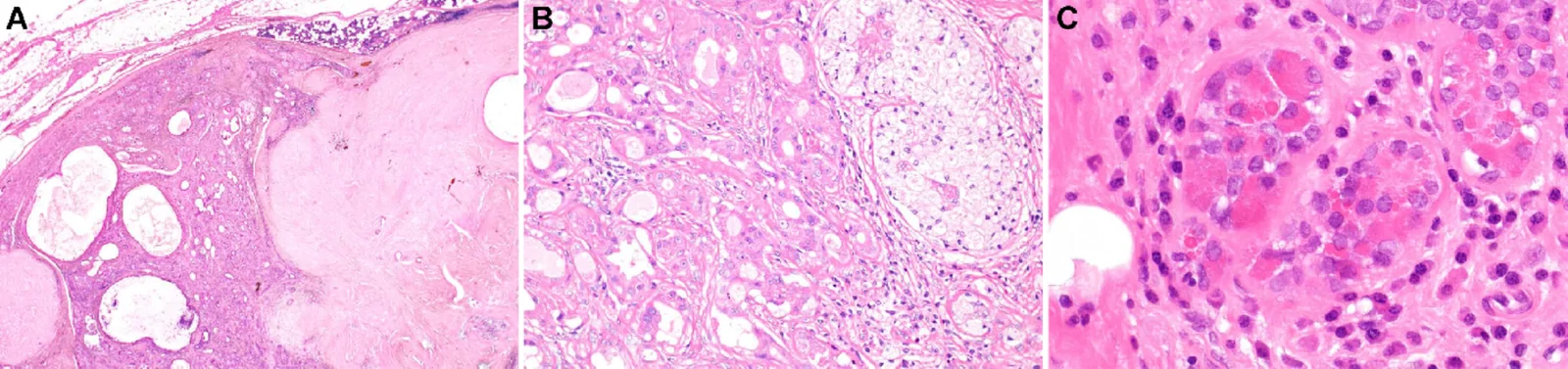
Histopathology of sclerosing polycystic adenoma. **a** Low-power hematoxylin and eosin (H&E) staining shows a well-defined tumor with extensive stromal fibrosis and hyalinization. **b** Medium-power H&E staining shows cystically dilated ducts lined by cells with diverse cytologic appearances, including eosinophilic and foamy changes. **c** High-power H&E staining shows acinar cells containing coarse hyper-eosinophilic granules. Images courtesy of Dr. Masato Nakaguro and Dr. Haruna Yagi, Department of Pathology and Laboratory Medicine, Nagoya University Graduate School of Medicine, Aichi, Japan. Adapted from the study by Yagi and Nakaguro [4]. Reproduced with permission from the publisher (Bunkodo Co., Ltd.)

SPA is a well-circumscribed tumor composed of large acinar cells with brightly eosinophilic intracytoplasmic granules and variably sized, cystically dilated ducts lined by foamy, vacuolated, or apocrine cells [1]. Representative pathological findings of SPA are shown in Fig. 2.

#### Background of classification and clinical presentation

SPA was first described by Smith et al. in 1996 in a series of nine patients from the Armed Forces Institute of Pathology [23]. As it resembles fibrocystic disease or sclerosing adenosis of the breast, researchers initially termed it sclerosing polycystic adenosis [23]. Since then, approximately 100 cases have been reported, mainly as case reports and small series [23–34]. For many years, the biologic nature of SPA remained controversial; it was considered reactive or inflammatory rather than neoplastic [23]. In the fourth edition of the WHO classification, SPA was included among non-neoplastic epithelial lesions [21, 22, 35, 36]. Subsequent molecular evidence supported its neoplastic nature [16, 17, 37], and SPA is now classified as a benign epithelial tumor in the fifth WHO classification [1–4, 22].

SPA occurs over a wide age range (7–84 years; mean, approximately 40 years) and shows a slight female predominance [1–4, 23–36]. Approximately 70% of SPAs arise in the parotid gland; occasional cases occur in the submandibular gland or intraoral minor salivary glands [1–4, 23–36]. Patients typically present with a painless, slowly growing mass, although pain occurs in some cases [1–4, 23–36].

#### Pathology

Macroscopically, SPA is a well-circumscribed, usually unencapsulated, firm or rubbery mass with multiple small cysts, typically 1–3 mm in diameter. The cut surface ranges from whitish tan to yellow-white. Reported tumor size ranges from 1 to 12 cm (mean, approximately 30 mm) [1–4, 23, 24, 27, 30, 31, 33, 34, 36].

Histologically, SPA is well-circumscribed and is unencapsulated or partially encapsulated [1–4, 23, 24, 27, 30, 31, 33, 34, 36]. It comprises multiple irregular lobules containing ducts, myoepithelial cells, and acini embedded in dense sclerotic or hyalinized stroma [1–4, 23, 24, 27, 30, 31, 33, 34, 36]. Chronic stromal inflammatory infiltrates range from sparse to dense [1–4, 23, 24, 27, 30, 31, 33, 34, 36]. The ducts are often cystically dilated and lined by flattened to cuboidal cells. The ductal epithelium may show vacuolated, apocrine, foamy, mucous, squamous, or sebaceous-like metaplasia [1–4, 23, 24, 27, 30, 31, 33, 34, 36]. The acinar cells usually contain coarse eosinophilic granules that are periodic acid-Schiff-positive and diastase-resistant; these granules likely represent altered zymogen granules and are a hallmark feature of SPA [1–4, 23, 24, 27, 30, 31, 33, 34, 36]. Intraductal epithelial proliferations range from minimal change to florid mammary-type hyperplasia with solid, micropapillary, or cribriform architecture. These proliferating cells may show dysplasia ranging from mild atypia to severe atypia resembling carcinoma in situ [1–4, 23, 24, 27, 30, 31, 33, 34, 36]. Although dense stromal sclerosis or fibrosis is characteristic, some cases contain foci of mature adipose tissue or abundant lipomatous stroma [2, 24, 27, 32]. The histologic appearance of SPA is variable, and some cases lack one or more typical features, such as cyst formation, stromal fibrosis, or apocrine change [26, 27].

Immunohistochemically, each cellular component generally shows the expected phenotype. Myoepithelial markers, including α-smooth muscle actin (SMA), p40, calponin, and S100, help demonstrate the intact myoepithelial layer around most ducts and acini [1–4, 23, 24, 27, 30, 31, 33, 34, 36].

Next-generation sequencing studies by Bishop et al. [16] identified phosphatidylinositol 3-kinase (PI3K) pathway mutations, most often involving *PTEN*, in all examined SPAs, including those without intraductal proliferation. These findings support the neoplastic nature of SPA and suggest a relationship to apocrine-type intraductal carcinoma or salivary duct carcinoma. PTEN immunohistochemistry showed loss of expression in ductal and acinar cells, with retained expression in myoepithelial cells, suggesting that myoepithelial cells may be non-neoplastic [16].

The histologic differential diagnosis includes benign and malignant SGTs and reactive lesions, such as pleomorphic adenoma (PA); IgG4-related sialadenitis; polycystic disease of the salivary gland; salivary carcinoma, not otherwise specified; low-grade mucoepidermoid carcinoma (MEC); and acinic cell carcinoma [1–4, 23, 27, 36].

#### Imaging findings

Large imaging series of SPA are unavailable, and current knowledge is based mainly on case reports. Agarwal et al. [38] described magnetic resonance imaging (MRI) findings of a left parotid SPA as a well-defined, smoothly marginated lesion with low signal intensity on both T1-weighted imaging (T1WI) and T2-weighted imaging (T2WI). Contrast-enhanced MRI showed predominantly peripheral enhancement with weaker central enhancement. Uemura et al. [28] reported a left parotid SPA that appeared hypoechoic, with heterogeneous internal echotexture on ultrasonography. Contrast-enhanced computed tomography (CT) showed heterogeneous enhancement. MRI showed low signal intensity on T1WI and mixed low- and high-signal intensity on T2WI, with heterogeneous enhancement after contrast administration.

As SPA is histologically diverse, its imaging appearance is likely variable. Saito et al. [32] reported a case with abundant fat at the periphery of the lesion, suggesting that SPA may be considered in the differential diagnosis of fat-containing SGTs, although this pattern appears rare. Yee et al. [29] reported a case with grossly visible large cystic components, indicating that SPA can also mimic a macroscopically cystic salivary lesion.

**Fig. 3:**
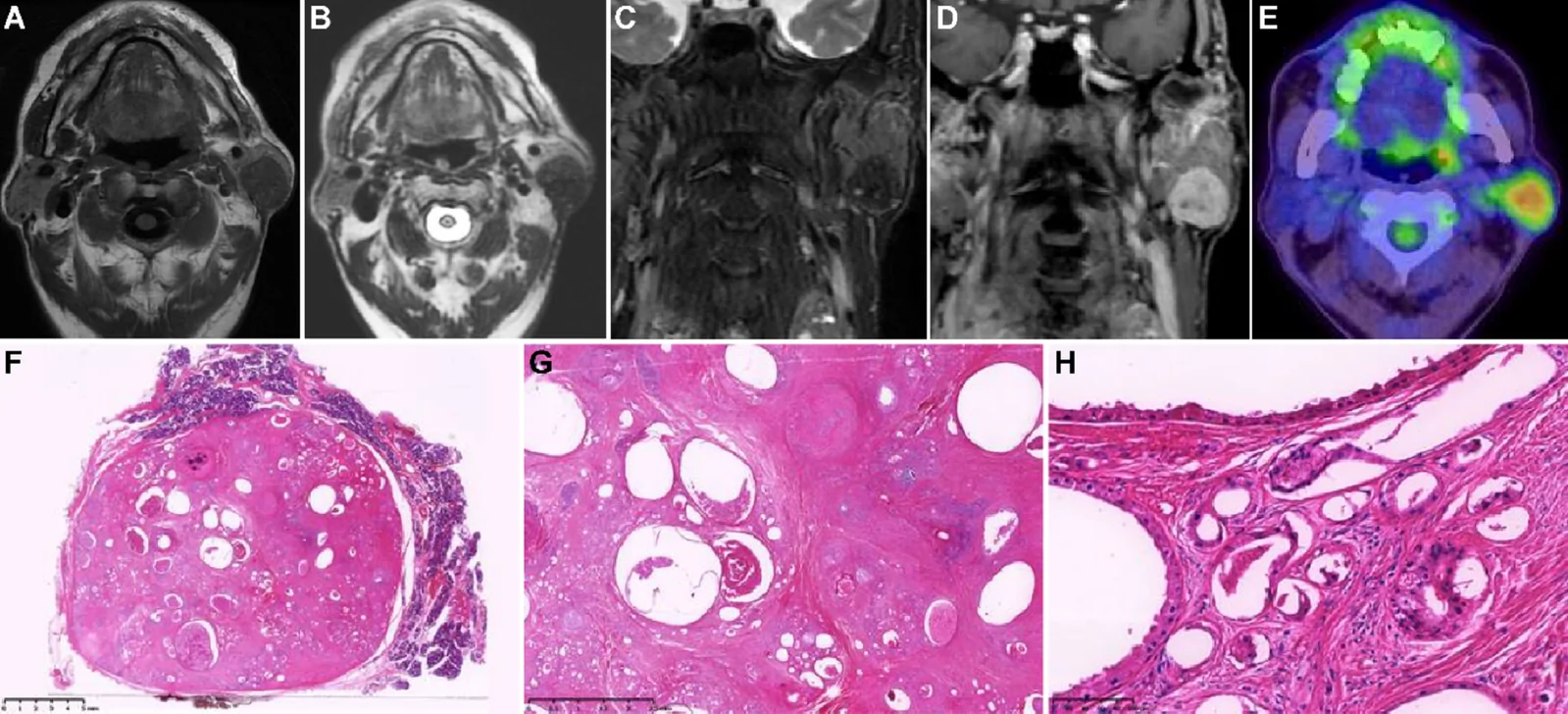
Imaging and histopathologic findings of sclerosing polycystic adenoma in a man in his 60 s. **a** T1-weighted MR image shows a mass with lower signal intensity than the surrounding parotid tissue. **b** T2-weighted MR image shows a mass with lower signal intensity than the surrounding parotid tissue. **c** Fat-suppressed coronal T2-weighted MR image shows a mass with lower signal intensity than the surrounding parotid tissue, with a tiny cyst visible as a small hyperintense focus. **d** Contrast-enhanced coronal MR image shows marked enhancement, with persistent nonenhancement of the tiny cyst. **e** FDG-PET/CT shows increased FDG uptake in the mass (SUVmax, 4.57). **f** Low-magnification view shows a well-defined 27 × 23-mm tumor in the left parotid gland with variably sized cystic spaces and stromal fibrosis or sclerosis (H&E staining). **g** Low-power H&E staining shows variably sized, cystically dilated ducts surrounded by dense collagenous and sclerotic stroma. **h** High-power H&E staining shows dilated ducts lined by epithelial cells with apocrine metaplasia. Images courtesy of Dr. Asako Yamamoto, Department of Radiology, Teikyo University Graduate School of Medicine. Abbreviations: FDG-PET/CT, fluorodeoxyglucose positron emission tomography/computed tomography; H&E staining, hematoxylin and eosin staining; MR, magnetic resonance; SUVmax, maximum standardized uptake

In our case of left parotid SPA (Fig. 3), the lesion was a well-defined, smoothly marginated, round mass. It showed lower signal intensity than the surrounding parotid gland on both T1WI and T2WI, with particularly low signal on T2WI, close to that of skeletal muscle. Small cystic foci with very high signal intensity were present on T2WI. No capsule was visible. Contrast-enhanced MRI showed marked enhancement except in the small cystic spaces, and no capsule-like enhancement was observed. Fluorodeoxyglucose positron emission tomography/computed tomography showed increased uptake with an SUVmax of 4.57. On radiologic-pathologic correlation, the dense sclerotic stroma with bands of hyalinized fibrosis likely contributed to the very low signal intensity on T2WI, and tiny cystic lesions within the mass corresponded to cystically dilated ducts.

### KC

#### Definition

**Fig. 4:**
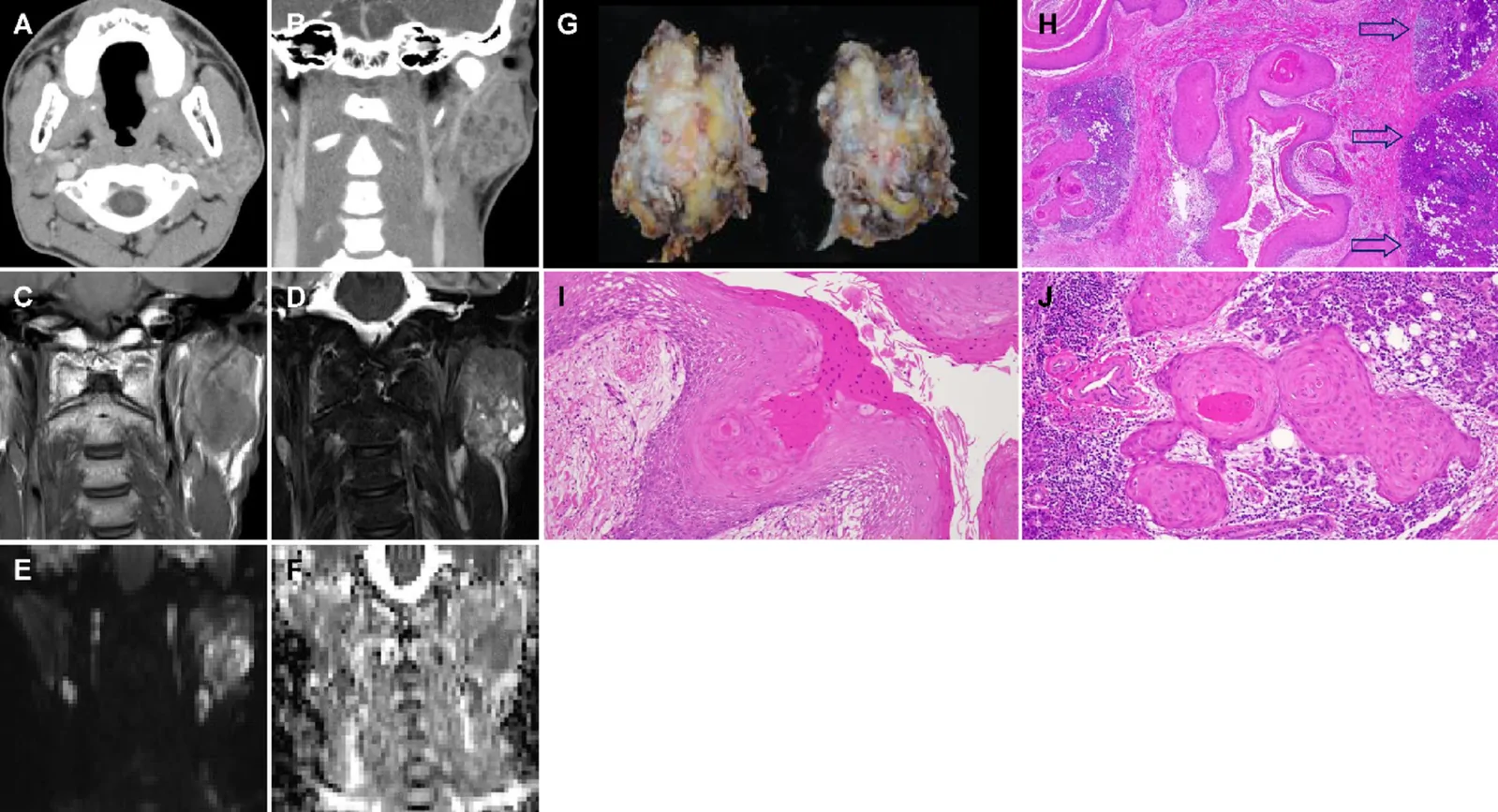
Imaging and histopathologic findings of keratocystoma in a teenage boy. **a** Contrast-enhanced axial CT image shows multiple ring-like enhancing foci within the mass. **b** Contrast-enhanced coronal CT image also shows ring-like enhancing foci within the mass. **c** Coronal T1-weighted MR image shows a mass with lower signal intensity than the surrounding parotid tissue. The boundary of the mass is ill-defined. No visible capsule is seen. **d** Fat-suppressed coronal T2-weighted MR image shows a mass with heterogeneous low-to-high signal intensity relative to the surrounding parotid tissue. Small cyst-like hyperintense structures are present within the lesion. The boundary of the mass is ill-defined. No visible capsule is seen. **e** Coronal diffusion-weighted MR image shows a mass with heterogeneous slightly high signal intensity. **f** The ADC value of the mass is 1.01 × 10^−3^ mm^2^/s on the ADC map. **g** Formalin-fixed specimen shows a white-to-gray cut surface. **h** Low-power H&E staining shows variably sized and shaped nests of keratinizing squamous epithelium embedded in fibrous stroma. Cystic spaces filled with lamellated keratin are also present in the central portion of the tumor nests. The boundary with the surrounding parotid tissue is clear, but no capsule is present (arrows). **i** High-power H&E staining shows a cystic space lined by well-differentiated squamous epithelium with parakeratosis and no granular cell layer. The squamous epithelium is regularly oriented from the outer basal cell layers to the inner keratotic layers. No cytologic atypia or nuclear pleomorphism is seen. **j** High-power H&E staining shows a single squamous cell nest with a preserved basement membrane. The surrounding parotid tissue shows chronic inflammatory cell infiltration, but no desmoplastic reaction is observed. Although the tumor nest appears irregularly shaped and complexly distributed within the parotid tissue, this should not be interpreted as invasive growth. Images courtesy of Dr. Yoichiro Kuwata, Department of Radiology, Nishi-Kobe Medical Center, and Dr. Maki Kanzawa, Department of Pathology, Nishi-Kobe Medical Center. Adapted from the study by Komatsu et al. [39]. This case was published previously [39]. Reproduced with permission from the publisher (Elsevier). Abbreviations: ADC, apparent diffusion coefficient; CT, computed tomography; H&E staining, hematoxylin and eosin staining; MR, magnetic resonance

KC is a benign SGT characterized by multicystic spaces lined by stratified squamous epithelium, keratotic lamellae, and focal solid epithelial nests [1]. Representative pathological findings of KC are shown in Fig. 4.

#### Background of classification and clinical presentation

KC was first described by Seifert et al. in 1999 and was initially regarded as a choristoma [40]. Nagao et al. reported two additional cases in 2002 and introduced the term keratocystoma [41]. Since then, sporadic cases have been reported [39, 42]. KC was not adopted as a distinct entity in the third or fourth WHO classification, although it was briefly mentioned in the differential diagnosis of squamous cell carcinoma (SCC) of the salivary gland in the third edition [43]. Subsequent molecular findings supported KC as a distinct benign tumor [20] and is now listed as a separate entity in the fifth WHO classification [1–4, 22].

According to Utsumi et al. [44], 21 cases, including probable cases, have been described in the English-language literature. Almost all reported tumors arose in the parotid gland [1–4, 20, 39–44]. Reported tumor size ranges from 8 to 40 mm (median, 25 mm) [1–4, 20, 39–44]. KC was initially thought to occur mainly in younger patients, but the reported age range has broadened to include ages extending from infancy to older than 70 years (median, 34 years) [1–4, 20, 39–44]. No clear sex predilection has been identified, and multiple or bilateral lesions have not been reported [1–4, 20, 39–44]. Clinically, KC typically presents as a slowly enlarging parotid mass [1–4, 20, 39–44]. Mild tenderness or facial nerve palsy related to tumor compression has rarely been described [39].

#### Pathology

Macroscopically, KC is a well-circumscribed, multicystic mass filled with whitish keratinous material, although published gross photographs are limited [39, 41, 42].

Histologically, KC comprises numerous variably sized cystic spaces haphazardly distributed throughout the lesion [1–4, 20, 39–44]. The cystic spaces are lined by well-differentiated keratinizing stratified squamous epithelium [1–4, 20, 39–44]. A fibrous capsule is absent [1–4, 20, 39–44]. The cystic spaces contain lamellated keratin with parakeratosis or orthokeratosis [1–4, 20, 39–44]. The epithelium lacks a granular cell layer and may form bud-like protrusions; skin adnexal structures are absent [1–4, 20, 39–44]. Solid nests of bland stratified squamous epithelium are present within fibrous stroma [1–4, 20, 39–44]. The tumor lacks sebaceous, follicular, oncocytic, and mucous cell differentiation [1–4, 20, 39–44]. Malignant features, including necrosis, invasion, increased mitotic activity, and intravascular growth, are absent [1–4, 20, 39–44]. In some cases, parotid duct epithelium gradually transitions to neoplastic squamous epithelium [20, 41, 44]. Stromal foreign body reaction to keratinous material and lymphoplasmacytic infiltration are common [1–4, 20, 39–44].

Immunohistochemically, the tumor cells resemble normal squamous epithelium, showing p63 positivity and negativity for myoepithelial markers, including SMA, calponin, and S100 [1–4, 20, 39–44]. The Ki-67 labeling index is low, and p53 shows a wild-type pattern [1–4, 20, 41, 44].

Bishop et al. [20] identified characteristic *RUNX2* rearrangements in KC by RNA sequencing, with confirmation by reverse transcription polymerase chain reaction or fluorescence in situ hybridization. *RUNX2* rearrangement, especially the *IRF2BP2::RUNX2* fusion, has not been identified in other SGTs and is considered characteristic of KC [1–4, 20].

The histological differential diagnosis includes non-neoplastic and neoplastic squamous lesions, including SCC, MEC, metaplastic Warthin tumor, PA with squamous differentiation, dermoid cyst, epidermoid cyst, and necrotizing sialometaplasia [1–4, 20, 41, 44].

#### Imaging findings

Imaging reports of KC are scarce. To date, only one well-documented case with a confirmed *RUNX2* rearrangement has been described in detail [39]. In 2018, Komatsu et al. [39] reported a left parotid KC appearing as a multicystic mass on CT and MRI. Contrast-enhanced CT showed multiple small ring-like enhancing foci within the lesion. On T1WI, the mass was hypointense, whereas on T2WI, it showed heterogeneous hypo- to hyperintensity. Small cystic spaces with very high signal intensity were present on T2WI. On both sequences, the boundary of the mass was ill-defined. No capsule was visible.

Theoretically, since KC contains keratotic lamellae, it is expected to show restricted diffusion on diffusion-weighted imaging. Diffusion-weighted MRI with apparent diffusion coefficient measurements for checking restricted diffusion might be a promising diagnostic approach for KC, although its value awaits further evaluation in a larger number of cases.

### SDA

#### Definition

**Fig. 5:**
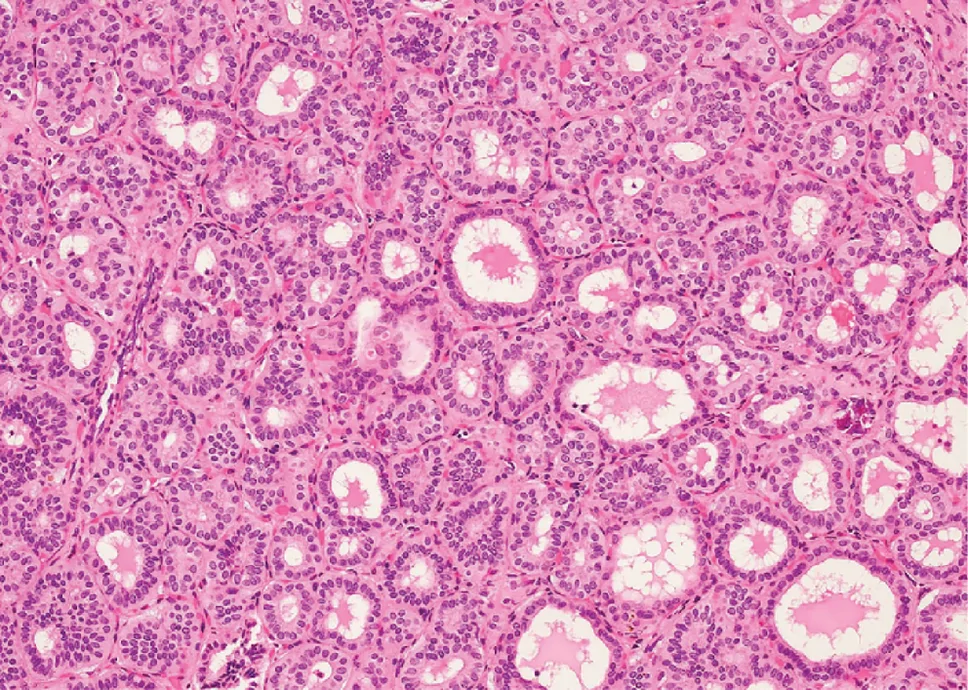
Histopathology of striated duct adenoma. H&E staining shows tumor cells with eosinophilic cytoplasm forming closely packed ducts and tubules in a back-to-back arrangement, with minimal intervening stroma. Image courtesy of Dr. Masato Nakaguro and Dr. Haruna Yagi, Department of Pathology and Laboratory Medicine, Nagoya University Graduate School of Medicine, Aichi, Japan. Adapted from the study by Yagi and Nakaguro [4]. Reproduced with permission from the publisher (Bunkodo Co., Ltd.). Abbreviations: H&E staining, hematoxylin and eosin staining

SDA is a benign SGT composed of ducts that resemble normal striated ducts and are lined by a single layer of luminal cells with minimal intervening stroma [1]. Representative pathological findings of SDA are shown in Fig. 5.

#### Background of classification and clinical presentation

Weinreb et al. [45] published the first formal clinicopathologic study of this tumor in 2010. The authors reported six cases and used the term SDA for a salivary gland ductal adenoma with striated differentiation [45]. For many years, SDA was not listed as a distinct entity in the WHO classification and was regarded as a variant of canalicular adenoma (CAD) [21, 22]. In the fourth WHO classification, SDA was briefly mentioned in the differential diagnosis of CAD, with the difference between CAD and the reported cases of SAD regarded as arbitrary [21]. Subsequent molecular evidence supported SDA as a distinct benign tumor [19] and is now listed as a separate entity in the fifth WHO classification [1–4, 22].

SDA is rare; according to Martins-Chaves et al. [46], only 20 cases have been documented in the literature. SDA occurs in both the major and minor salivary glands but is commonly seen in the parotid gland [1–4, 19, 45–48]. Tumor size ranges from 0.5 to 3.0 cm (mean, 1.5 cm), although Ito et al. [47] reported an unusually large hemorrhagic tumor measuring 6 cm. Reported patients were adults aged 47–78 years, with a slight female predominance [1–4, 19, 45–48]. Clinically, SDA presents as a slowly enlarging, painless mass [1–4, 19, 45–48].

#### Pathology

Macroscopically, SDAs have been described as solid masses with yellow cut surfaces, although gross descriptions remain limited [47, 48].

Histologically, SDAs are well-circumscribed, encapsulated tumors composed of closely packed small ducts separated by minimal stroma [1–4, 19, 45–48]. Small cysts may be present. The stroma may be highly vascular, and some tumors contain staghorn-like vessels [1–4, 19, 45–48]. The ducts are lined by a single layer of columnar cells with eosinophilic cytoplasm, distinct cell borders, and intracytoplasmic striations [1–4, 19, 45–48]. Lumina may contain eosinophilic colloid-like material or red blood cells [1–4, 19, 45–48]. Some cases show stromal hyalinization and may resemble oncocytoma with oncocytosis [1–4, 19, 45, 46]. Striated duct hyperplasia may be present in adjacent parotid tissue [1–4, 19, 45, 46]. Duct caliber is similar to that of normal striated ducts, with some ducts showing cystic dilatation up to 1 mm [1–4, 19, 45–48]. Fat cells may be present focally or diffusely [1–4, 19, 45–48]. The nuclei are uniform and oval and may show grooves or pseudoinclusions resembling those seen in papillary thyroid carcinoma [1–4, 19, 45–48].

Immunohistochemically, SDA is diffusely positive for CK7 and S100 [1–4, 19, 45–48]. It lacks true myoepithelial differentiation and is usually negative for calponin, p63, SMA, smooth muscle myosin heavy chain, and other basal or myoepithelial markers [1–4, 19, 45–48]. Some cases may show focal isolated staining for these markers; however, this finding should not be interpreted as evidence of a true basal or myoepithelial layer [1–4, 19, 45–48]. Markers of thyroid differentiation, including thyroid transcription factor 1 and thyroglobulin, are negative [1–4, 45–48].

Rooper et al. [19] identified characteristic *IDH2* p.R172X mutations in SDA, supporting its classification as a distinct entity separate from CAD. This mutation has not been identified in other SGTs and is regarded as characteristic of SDA [1–4, 19].

The main histologic differential diagnosis is CAD [1–4, 19, 45, 46]. Other differential diagnoses include IDA/IDH and basal cell adenoma (BCA); oncocytoma should also be considered because SDA cells may have eosinophilic cytoplasm [1–4, 19, 45, 46].

#### Imaging findings

Imaging reports of SDA are scarce. Only two case reports have described imaging findings [47, 48]. Ito et al. [47] reported a left parotid SDA with intratumoral hemorrhage; however, only a fat-suppressed T2-weighted magnetic resonance image was provided, and thus the complete MRI appearance was not defined. Monarchi et al. [48] reported an SDA of the palate that showed marked enhancement on contrast-enhanced CT. In both reports, the authors suggested that the tumor’s rich stromal vascular network may have contributed to intratumoral hemorrhage or strong enhancement.

### IDA/IDH

#### Definition

**Fig. 6:**
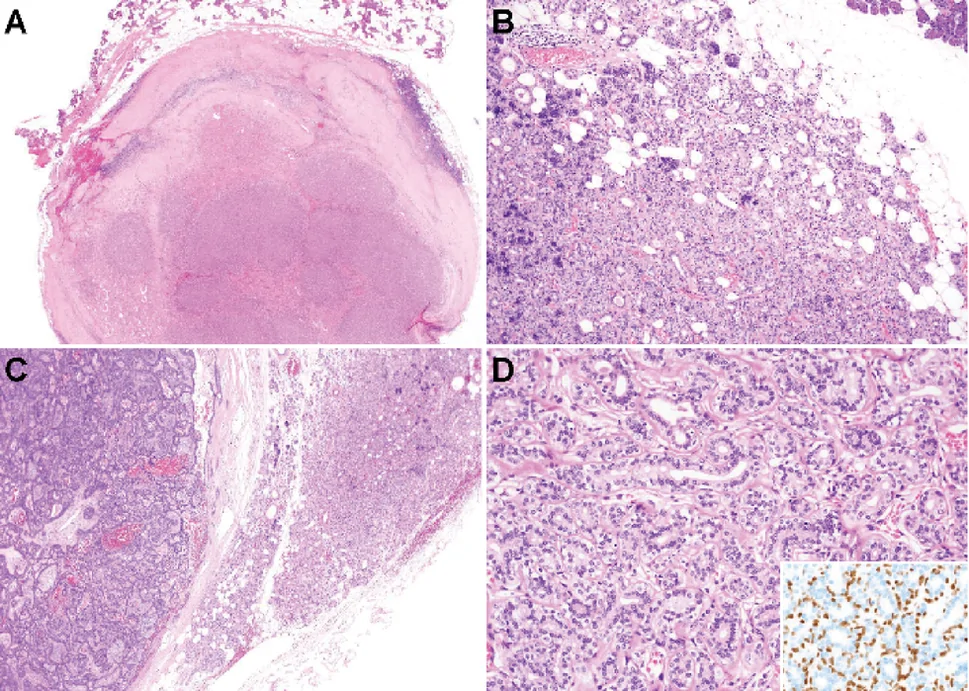
Histopathology of intercalated duct adenoma and hyperplasia. **a** Intercalated duct adenoma (IDA) appears as a well-defined nodular lesion on hematoxylin and eosin (H&E) staining. **b** Intercalated duct hyperplasia (IDH) lacks a capsule and intermingles with normal salivary gland tissue on H&E staining. **c** H&E staining shows IDH on the right, adjacent to basal cell adenoma on the left. **d** H&E staining shows dense proliferation of small-caliber ducts composed of cuboidal epithelial cells. The surrounding myoepithelial cells are difficult to identify on routine H&E staining. The inset shows p63 immunostaining highlighting myoepithelial cells around all ducts. Image courtesy of Dr. Masato Nakaguro and Dr. Haruna Yagi, Department of Pathology and Laboratory Medicine, Nagoya University Graduate School of Medicine, Aichi, Japan. Adapted from the study by Yagi and Nakaguro [4]. Reproduced with permission from the publisher (Bunkodo Co., Ltd.)

IDA/IDH refers to ductal proliferations resembling bilayered intercalated ducts composed of epithelial and myoepithelial cells [1]. Representative pathological findings of IDA/IDH are shown in Fig. 6.

#### Background of classification and clinical presentation

The first comprehensive report on intercalated duct lesions (IDLs) was published by Yu and Donath in 2001, in which 13 cases were described under the term “adenomatous ductal proliferation/hyperplasia” [49]. Since this seminal report, several case series and reports have been published [50–58], with the largest series comprising 32 patients with 34 lesions published by Weinreb et al. [50]. Overall, fewer than 80 cases of IDLs have been reported to date [21]. IDLs are uniform proliferations of cuboidal ductal cells encircled by myoepithelial cells, resembling normal intercalated ducts of the salivary duct system [1–4, 18, 49–58]. They represent a morphologic spectrum from IDH to IDA [1–4, 18, 49–58]. Their biological nature was debated for many years. The term IDL has been used as an umbrella term for both the neoplastic adenoma and non-neoplastic hyperplastic forms [50]. In the fourth WHO classification, IDLs were included among non-neoplastic epithelial lesions [21]. A subset of IDLs has been reported to coexist and share molecular features with other SGTs, most commonly BCA and epithelial-myoepithelial carcinoma (EMC) [11, 18, 50]. In the fifth WHO classification, IDLs are included among benign SGTs as IDA/IDH [1–4, 22].

Approximately 85% of cases arise in the parotid gland, followed by the submandibular gland (11%) and intraoral minor salivary glands (4%) [1, 50]. Most lesions are incidental findings identified with other salivary gland lesions, such as BCA or EMC [1–4, 11, 18, 49–58]. IDA/IDH occurs across a wide age range (19–80 years; mean, 53.8 years), with a male-to-female ratio of 1:1.7 [1, 50].

No single tumor-specific genetic alteration has been established; however, a subset of IDAs harbors *HRAS* mutations, whereas IDH often harbors *CTNNB1* mutations [18]. Based on these findings, intercalated duct lesions have been proposed as precursor lesions of BCA or EMC [1–4, 11, 18, 50, 53].

#### Pathology

Macroscopically, IDA/IDH is usually not apparent because most lesions are microscopic, with most measuring less than 5 mm [1–4, 18, 49–58].

Histologically, intercalated duct lesions consist of tightly packed small cuboidal ducts with attenuated myoepithelial cells and little to no intervening stroma; adjacent acini may be present [1–4, 18, 49–58]. Weinreb et al. proposed separating these lesions into adenoma and hyperplasia [50]. IDH lacks a capsule, has an irregular border, and merges with adjacent salivary tissue, often entrapping acinar cells at the periphery [50]. IDA is well circumscribed, encapsulated, and sharply demarcated from adjacent salivary tissue [50]. Hybrid lesions with both hyperplastic and adenomatous areas have also been reported [18, 50, 56, 57]. The myoepithelial cells are small and have scant cytoplasm, making them difficult to identify on routine hematoxylin–eosin sections [1–4, 18, 49–58]. Immunohistochemistry with myoepithelial markers helps demonstrate the thin myoepithelial layer around all ducts [1–4, 18, 49–58].

Immunohistochemically, the ductal epithelial cells are positive for CK7 and often for S100 and estrogen receptor, whereas the myoepithelial cells are positive for myoepithelial markers, including p63, calponin, and CK14 [1–4, 18, 49–58].

The histologic differential diagnosis includes BCA, SDA, and CAD [1–4, 45, 50, 58].

#### Imaging findings

Detailed imaging descriptions of IDA/IDH are unavailable because these lesions are usually microscopic. Mok et al. [56] reported one pathologically diagnosed hybrid-type lesion, but its MRI appearance was indistinguishable from that of an intraparotid lymph node.

## Conclusion

This review summarizes the four benign SGTs newly listed in the fifth WHO classification, describing their clinicopathologic features and the limited imaging findings reported to date. Molecular pathology of SGTs has greatly advanced in recent years, allowing better understanding and classification of SGTs. As a result, SPA and IDA/IDH have been added to the benign epithelial tumors category from the non-neoplastic epithelial lesions category in the fifth WHO classification. Molecular testing has led to the discovery of novel tumor types, including SDA and KC, which have been established in the fifth WHO classification. These four new entities are defined by characteristic molecular alterations and histopathologic features.

As imaging findings for these rare tumors remain incompletely defined, familiarity with the fifth WHO classification, relevant immunohistochemical markers, and genetic alterations is important for radiologists, and radiologic-pathologic correlation remains central to accurate diagnosis.
